# Breaking the feed forward inflammatory cytokine loop in the tumor microenvironment of PDGFB-driven glioblastomas

**DOI:** 10.1172/JCI175127

**Published:** 2023-11-15

**Authors:** C. Ryan Miller, Anita B. Hjelmeland

**Affiliations:** 1 Department of Pathology, Division of Neuropathology and O’Neal Comprehensive Cancer Center, and; 2 Department of Cell, Developmental, and Integrative Biology and O’Neal Comprehensive Cancer Center, University of Alabama at Birmingham, Birmingham, Alabama, USA.

## Abstract

Glioblastoma (GBM) tumor-associated macrophages (TAMs) provide a major immune cell population contributing to growth and immunosuppression via the production of proinflammatory factors, including IL-1. In this issue of the *JCI*, Chen, Giotti, and colleagues investigated loss of *ll1b* in the immune tumor microenvironment (TME) in GBM models driven by PDGFB expression and *Nf1* knockdown. Survival was only improved in PDGFB-driven GBM models, suggesting that tumor cell genotype influenced the immune TME. IL-1β in the TME increased PDGFB-driven GBM growth by increasing tumor-derived NF-κB, expression of monocyte chemoattractants, and increased infiltration of bone marrow–derived myeloid cells (BMDMs). In contrast, no requirement for IL-1β was evident in *Nf1*-silenced tumors due to high basal levels of NF-κB and monocyte chemoattractants and increased infiltration of BMDM and TAMs. Notably, treatment of mice bearing PDGFB-driven GBM with anti–IL-1β or an IL1R1 antagonist extended survival. These findings suggest that effective clinical immunotherapy may require differential targeting strategies.

## The RCAS/*Ntv-a* system to model glioblastoma subtypes

The promise of cancer immunotherapy requires understanding the interactions between neoplastic cells and the immune tumor microenvironment (TME). In the primary brain tumor glioblastoma (GBM), tumor-associated macrophages (TAMs) originate from infiltrating bone marrow–derived myeloid cells (BMDMs) or resident microglia and can constitute 50% of the cells within the tumor (1). Considering their high prevalence, TAMs are thought to be a major immune cell population contributing to GBM growth and immunosuppression that could be therapeutically targeted.

To better understand the interaction between GBM cells and TAMs, the Hambardzumyan laboratory has used the replication-competent ASLV long terminal repeat (LTR) with a splice acceptor (RCAS) tumor virus A (TVA) system. In RCAS-TVA–based mouse models of human cancers, cell-specific expression of avian retroviral receptor permits infection by avian retroviruses that carry oncogenes and/or gene-silencing components for knocking down tumor suppressors (2, 3). By employing different genetic alterations that are associated with distinct transcriptional signatures, researchers can model distinct tumor subtypes (4). In human GBM, proneural, mesenchymal, and classical transcriptional subtypes have been identified, but all can be present within a single patient (5, 6). Moreover, these subtypes are associated with anatomically distinct biomes within the tumor (7). In this issue of the *JCI*, Chen, Giotti, et al. used RCAS vectors to elevate PDGFB or silence the tumor suppressor genes *Nf1*, *Trp53*, and *Pten*. This strategy modeled proneural GBM (PDGFB-driven GBM) and mesenchymal GBM (*Nf1*-silenced GBM), respectively (8). Both models used Nestin promoter–driven TVA to permit genetic alteration of neural stem cells, which have been proposed as a GBM cell of origin (9). While these genetically defined models simplify the issue of intratumoral heterogeneity, they also provide the opportunity for exploring how different genetic alterations affect GBM growth in vivo.

## A protumorigenic role for IL-1B in PDGFB-driven GBM

Using the RCAS-TVA system in immunocompetent mice that also lack immune-related genes can reveal roles for genes of interest in the various cell types making up the GBM TME. Chen, Giotti, et al. focused on IL-1, a cytokine encoded by *IL1A* and *IL1B* (8). IL-1 regulates GBM growth through affecting the tumor immune landscape (8, 10, 11). IL-1 is a known proinflammatory factor that is implicated in promoting tumor growth in several cancer types, including GBM (10–16). While more precise roles in GBM remained to be determined, IL-1 was hypothesized to be important here because it is secreted by BMDM cells, which can differentiate into TAMs. Thus, Chen, Giotti, et al. generated PDGFB-driven and *Nf1-*silenced GBM models in mice deficient in *Il1b* or both *Il1a* and *Il1b* (8).

**Figure 1:**
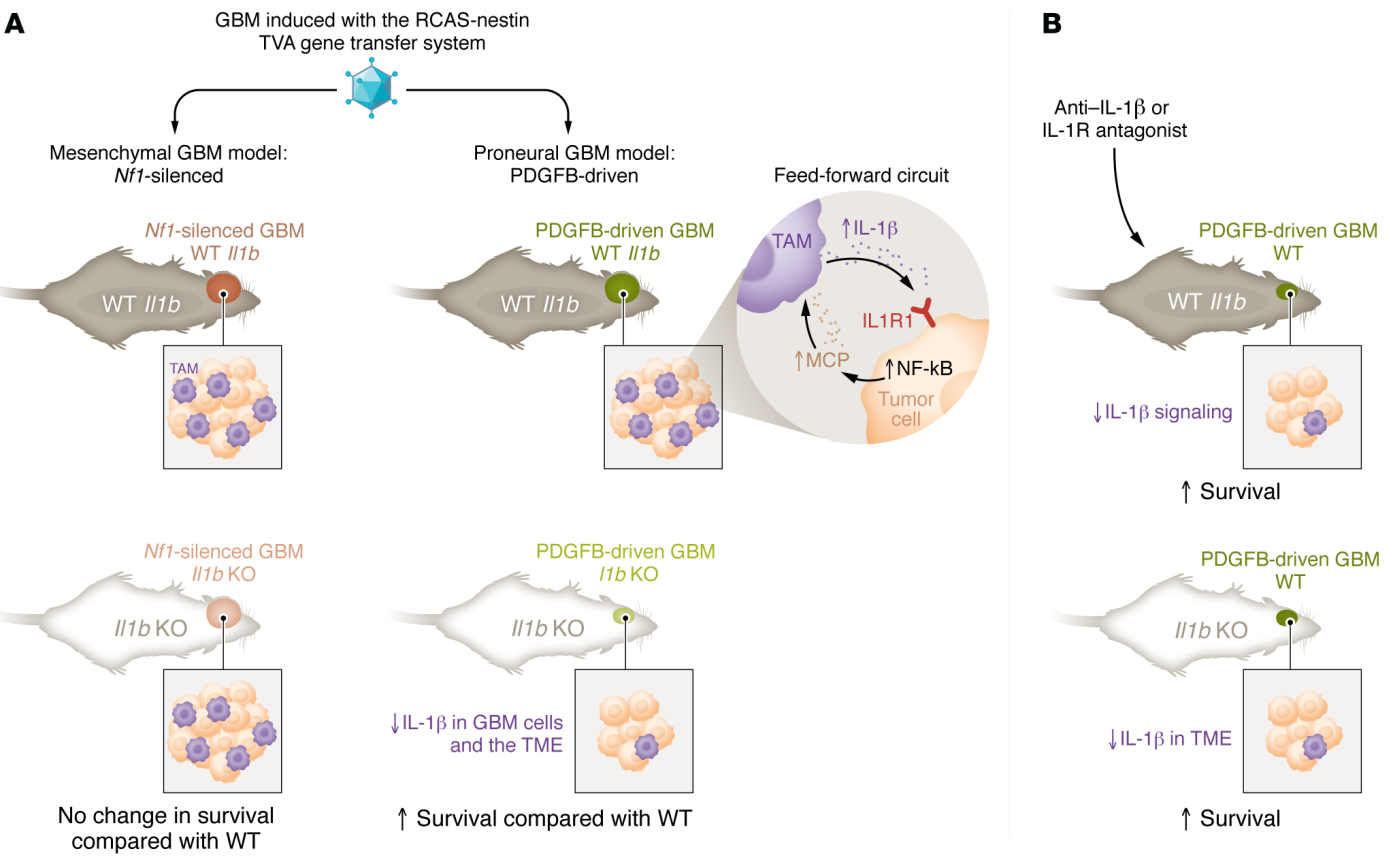
PDGFB-driven GBM cells and TAMs that express IL-1β establish a feed-forward loop. (**A**) Chen, Giotti, et al. determined that the requirement for IL-1β in GBM growth differed based on tumor cell genotype. The RCAS/*Ntv-a* system was used to drive GBMs based on elevated PDGFB expression or *Nf1* knockdown in a genetic background with or without IL-1β knockout. Survival of mice with PDGFB-driven but not *Nf1-*silenced GBMs was increased in *Il1b*-knockout mice. In PDGFB-driven GBMs, IL-1β stimulated NF-κB activity and MCP production to increase BMDM infiltration. In contrast, high basal levels of NF-kB activity in *Nf1*-silenced GBMs drove growth via MCP production and BMDM infiltration. (**B**) IL-1β specifically from the TME drives tumor growth. Targeting of IL-1β or its receptor IL1R1 improved the survival of mice bearing PDGFB-driven GBMs. Similarly, *Il1b* loss in the TME, but not GBM, cells limited GBM growth and increased survival in mice.

In the PDGFB-driven GBM model, loss of *Il1b* increased survival in male and female mice (8). However, male survival (63 days) was substantially less than that of females (81 days) in *Il1b* knockout, while no sex differences were evident in control, WT mice (48 versus 47 days, respectively). The survival benefit from loss of *Il1b* was due to contributions from the TME (Figure 1). Specifically, mice orthotopically injected with PDGFB-driven GBM cells, which were generated in *Il1b* WT mice, showed extended survival in the context of *Il1b* knockout (8). This microenvironmental role for IL-1β was consistent with immunofluorescence studies using GBM sections, which demonstrated that IL-1β expression was highest in regions costaining for the microglia/macrophage marker IBA1 and with single-cell RNA-Seq data demonstrating high *Il1b* in BMDMs rather than tumor cells. When the immune landscape of these tumors was profiled, the percentage of BMDMs was decreased in *Il1b*-null mice (8). Evaluation of myeloid cell subsets demonstrated that there were decreases in monocytes, macrophages, and neutrophils in PDGFB-driven GBMs generated in *Il1b*-knockout mice compared with subsets in GBMs that were generated in WT controls. In addition, exhausted CD8^+^ T cells were decreased in *Il1b-*knockout mice. To explore the mechanism through which BMDM infiltration could be reduced with *Il1b* loss, levels of monocyte chemoattractant proteins (MCPs), including MCP1 (aka CCL2), MCP2 (aka CCL8), and MCP3 (aka CCL7), were elevated (8). MCPs are chemokines that promote monocyte migration and infiltration. MCP levels were elevated in PDGFB-driven GBM compared with normal brain, and treatment of these tumor cells with IL-1β increased MCP expression concomitant with increased NF-κB activation via phosphorylation. IL-1β was elevated in BMDMs when these cells were cocultured with organotypic slices of PDGFB-driven GBM-bearing mouse brains (8). Thus, the data suggest a feed-forward loop in which PDGFB-driven GBM cells express MCPs that drive BMDM infiltration and IL-1β expression, further promoting tumor growth.

## *Nf1*-silenced GBM growth did not require *Il1b*

In contrast with the results with the PDGFB-driven GBM model, there was no difference in survival in either males or females when *Nf1*-silenced GBMs were initiated using the RCAS-TVA system in *Il1b*-knockout versus control, WT mice (8). There were also no notable changes in monocyte infiltration into these tumors in mice with or without *Il1b* knockout. These findings are reminiscent of data in syngeneic GBMs indicating that PDGFB-, but not Ras-driven, GBMs were sensitive to TAM targeting via inhibition of colony stimulating factor 1 receptor (CSF1R), which is a potent regulator of myeloid cell growth and differentiation (17). While the data of Chen, Giotti, et al. indicated an IL1-β–independent mechanism for regulating BMDM infiltration in the *Nf1*-silenced tumors, data from The Cancer Genome Atlas (TCGA) showed levels of *IL1B* (and *IL1A*) were higher in human mesenchymal GBM, a finding consistent with elevated levels of *Il1b* in murine *Nf1*-silenced versus PDGFB-driven GBM models (8). This result was likely due to IL-1β expression in TAMs, which are known to be increased in perivascular and perinecrotic mesenchymal regions of human GBMs (18). To define mechanisms contributing to BMDM infiltration, Chen, Giotti, et al. evaluated whether the basal elevation of NF-κB signaling in *Nf1*-silenced GBMs contributed to MCP production. NF-κB phosphorylation and activation was higher in *Nf1*-silenced than PDGFB-driven GBMs, but was not further activated by IL-1β treatment. Inhibition of IκBα phosphorylation/NF-κB signaling with BAY 11-7082 was sufficient to reduce levels of secreted MCP1-3 (8). Together, these data suggest that increased NF-κB activity in *Nf1*-silenced GBM cells leads to elevated MCP levels, which promote infiltration of BMDMs expressing *Il1b*.

When either PDGFB-driven or *Nf1-*silenced GBMs were initiated, there was no survival benefit with the loss of *Il1a* in addition to *Il1b* (8). In fact, the survival extension observed in *Il1b-*knockout mice bearing PDGFB-driven GBMs was completely lost when *Il1a* was also deleted. These results suggested an antitumorigenic role for *Il1a* in these tumors, and knockout of *Il1a* alone did lead to increased GBM growth (8). While the mechanisms through which IL-1α affects tumor biology and whether its effects require IL-1β inhibition remain to be fully determined, Chen, Giotti, et al. found that tumors in *Il1a-* and *Il1b-*knockout mice had reduced microglia in association with decreased microglia proliferation, which was not observed in mice lacking *Il1b* alone. Data also suggested that the neural stem cell–like GBM stem cell (GSC), also known as brain tumor-initiating cell (BTIC), fraction that is associated with therapeutic resistance may be enriched with knockout of *Il1a* and *Il1b* compared with loss of *Il1b* alone (8). Further functional experiments are needed to explore this possibility.

## Preclinical studies demonstrate the benefit of targeting IL-1B/IL1R1

To translate their findings toward the clinic, Chen, Giotti, et al. treated mice bearing PDGFB-driven GBM with an antibody to IL-1β or an antagonist of its receptor IL1R1. When these treatments were administered directly into the brains of tumor-bearing mice, each was sufficient to increase survival and decrease the percentage of IBA1^+^ TAMs (8). Profiling of immune-related proteins with NanoString GeoMx indicated that anti–IL-1β–treated tumors had increased levels of granzyme B, a serine protease important for immune cell–mediated cytotoxicity of tumor cells. In addition, anti–IL-1β treatment reduced levels of the immune checkpoint programmed cell death protein 1 (PD-1) (8). Consistent with the notion that IL-1β could provide benefit for patients, higher levels of *IL1B* were associated with worse prognosis in human GBM data from TCGA.

## Future directions

While targeting TAMs via inhibition of IL-1β/IL1R1 signaling appears promising, there are many complexities that remain to be investigated. Importantly, the differential requirement for IL-1β in immune cell infiltration in the two GBM genotypes modeled in Chen, Giotti, et al. (8) indicates that intratumoral heterogeneity in patients must be considered. While GBM cells within a human tumor may have a main or dominant subtype and those subtypes are associated with differential immune cell infiltration, single-cell sequencing demonstrated the presence of multiple transcriptional subtypes within tumors of individual patients (5, 6). How interactions among GBM cells of different genotypes or transcriptional subtypes influence tumor growth and the prevalence and function of TAMs, including through IL-1β, remains to be further investigated. Furthermore, tumor cell subpopulations or subtypes can shift during or after standard-of-care therapies, which can reduce immune cell populations or alter cytokine production (19–22). Any of these effects are likely to affect GBM/immune cell interactions, so the timing of administration of treatments targeting TAMs needs to be carefully considered. For example, Chen, Giotti, et al. (8) highlight the fact that dexamethasone, a commonly used treatment for peritumor edema in GBM patients, is known to reduce IL-1β (23).

Sex-specific differences could also contribute to the ability to target the TME. In the studies presented in Chen, Giotti, et al. (8), median survival of both male and female mice harboring PDGFB-driven GBM increased with *Il1b* deletion, but female mice appeared to have a greater benefit (8). Indeed, a prior study including the mouse glioma cell line GL261 demonstrated that anti–IL-1β treatment increased the survival of female, but not male, mice bearing these tumors (11). While Chen et al. observed a sex-independent association of *IL1B* with poor patient prognosis (8), Bayik et al. found that *IL1B* was associated with poor prognosis in female, but not male, GBM patients using TCGA data separated based on the upper and lower quartiles of mRNA expression (11). Together, these data suggest that there could be sex-specific differences in IL-1β roles in GBM growth, including those that are sex hormone dependent. These potential differences and dependencies remain to be fully explored.

Patients with GBM are typically 65 years or older when diagnosed. While brain and immune system aging undoubtedly contribute to poor patient outcomes (24), age-related mechanisms are a highly understudied area of investigation. Experiments evaluating the contribution of the TME to GBM in this study (and the overwhelming majority of studies) involve injection of tumor cells into the brains of young adult, but not aged, mice. While working with aged mice (90-week-old mice are thought to correspond to 65-year-old individuals) is not practical for most research groups, it remains to be determined whether successful translation of preclinical trials in younger animals to the clinic will be hampered by age-related differences in immune system function.

In summary, Chen, Giotti, et al. (8) have produced a large body of in vitro and in vivo data, based on the RCAS/*Ntv-a* system, that demonstrates a protumorigenic role for IL-1β in PDGFB-driven GBM (Figure 1). Their research continues to build on increasing numbers of studies, suggesting the importance of understanding and targeting GBM-TAM interactions for improving patient survival.

## Version 1 11/15/2023

Electronic publication
